# Supplementary material for: Immune parameters of HIV susceptibility in the female genital tract before and after penile-vaginal sex
Source: Commun Med (Lond). 2022 May 27;2:60. doi: 10.1038/s43856-022-00122-7 (PMC9142516; doi:10.1038/s43856-022-00122-7)
Supplement: Supplementary file 1 — Description of Additional Supplementary Files [file 43856_2022_122_MOESM1_ESM.pdf]

Description of Additional Supplementary Files

File Name: Supplementary Data 1

Description: Source data analysed for the Results section and the main figures.
