# Supplementary material for: Immune parameters of HIV susceptibility in the female genital tract before and after penile-vaginal sex
Source: Commun Med (Lond). 2022 May 27;2:60. doi: 10.1038/s43856-022-00122-7 (PMC9142516; doi:10.1038/s43856-022-00122-7)
Supplement: Supplementary file 2 — Supplementary materials [file 43856_2022_122_MOESM2_ESM.pdf]

Title: Immune parameters of HIV susceptibility in the female genital tract before and after penile-vaginal sex

Avid Mohammadi[1], Sareh Bagherichimeh[1*], Yoojin Choi[2], Azadeh Fazel[1], Elizabeth Tevlin[3†], Sanja Huibner[1], Sara V Good[4], Wangari Tharao[3], Rupert Kaul[1]

Affiliations:

[1]Department of Medicine, University of Toronto, Toronto, Ontario, Canada.

[2]Department of Immunology, University of Toronto, Toronto, Ontario, Canada.

[3]Women's Health in Women's Hands Community Health Center, Toronto, Ontario, Canada.

[4]Department of Biology, University of Winnipeg, Winnipeg, Manitoba, Canada

* Present address: Department of Pathology and Laboratory Medicine at Schulich Medicine and Dentistry, University of Western, London, Ontario, Canada.

† Present address: Street Health Community Nursing, Toronto, Ontario, Canada.

Materials and Correspondence: Dr. Rupert Kaul and/or Mrs. Avid Mohammadi. Department of Medicine, University of Toronto, Medical Sciences Building #6356, Toronto, Ontario, Canada, M5S 1A8. Tel: (1-416) 978-8607; Fax: (1-416) 978-8765. Rupert.kaul@utoronto.ca ; Avid.mohammadi@mail.utoronto.ca

Conflict of interests: The authors declare no competing interests.

SUPLEMENTARY TABLE 1. Participant characteristic in women who had condomless sex based on circumcision status of the male partner

Characteristic	Uncircumcised (n=16)	Circumcised (n=13)
**Age (median; range)**	22 (18-33)	21 (19-33)
**Ethnicity**		
Asian (East or South Asian)	7 (43.8)	3 (23.1)
White	5 (31.3)	5 (38.5)
African-Caribbean and Black	1 (6.3)	1 (7.7)
Latin American	1 (6.3)	0
Middle Eastern	0	1 (7.7)
Mixed	2 (12.5)	3 (23.1)
**Contraceptive method**		
Oral contraceptive pill	5 (31.3)	5 (38.5)
Condom	3 (18.8)	1 (7.7)
Hormonal IUD	2 (12.5)	2 (15.4)
Copper IUD	1 (6.3)	2 (15.4)
NuvaRing	1 (6.3)	1 (7.7)
Condom and hormonal	0	1 (7.7)
None	4 (25)	1 (7.7)
**Vaginal douching**	2 (12.5)	2 (15.4)
**Bacterial vaginosis**	2 (12.5)	3 (23.1)
**Relationship length (months)**	11 (1-72)	24 (2-96)
**Time since last sex (days)**	3 (2-23)	4 (3-10)
**STI history**	2 (12.5)	1 (7.7)

SUPLEMENTARY TABLE 2. Baseline cytokine levels in semen based on circumcision status

Cytokines	Semen		p-value
	Uncircumcised (Median, range)	Circumcised (Median, range)	
**IP-10**	40,643.17 (1,499.81-77,457.33)	35,328.39 (14,591.45,328,349.53)	0.934
**MIG**	23,426.74 (471.79-38,100.08)	23,820.07 (4,233.68-333,954.49)	0.561
**MIP-1β**	630.23 (124.64-10,281.91)	720.32 (134.80-8,346.62)	0.678
**IL-8**	1,045.19 (159.87-18,608.36)	1,182.54 (265.75-12,110)	0.901
**MIP-3α**	1,159.77 (20.54-5,4791.11)	919.04 (394.81-19,212.50)	0.901
**IL-1α**	188.92 (79.46-2,695.76)	176.11 (72.10-64,079)	0.901
**IL-6**	8.11 (1.20-126.27)	8.42 (0.88-144.80)	0.506
**IL-17**	11.79 (2.43-258)	11.25 (4.20-644.94)	0.835
**IFNα2a**	39.63 (6.93-65.61)	39.04 (17.99-705.95)	0.771
**sE-cadherin**	290,649.64 (35,808.05-786,586.53)	266,298.45 (129,735.20-3,053,534.76)	0.739
**MMP9**	3,392.52 (461.92-1,246,997.34)	11,916.09 (164.55-723,728.70)	0.280

SUPLEMENTARY TABLE 3. Baseline cytokine levels in cervico-vaginal secretion based on circumcision status of the male partner

Cytokines	Cervico-vaginal secretion		p-value
	Uncircumcised (Median, range)	Circumcised (Median, range)	
**IP-10**	2,214.90 (108.91-66,407.83)	1,458.19 (9.90-8,181.43)	0.599
**MIG**	124.60 (49.40-5,393.74)	153.57 (15.82-940.45)	0.792
**MIP-1β**	307.07 (139-3,979)	518.92 (139-4,784.98)	0.303
**IL-8**	28,435.45 (364.73-37,935.93)	24,851.75 (2,082.25-42,978.93)	0.630
**MIP-3α**	766.29 (119.82-19,736)	793.03 (73.43-17,573.81)	0.965
**IL-1α**	23,507.52 (3,334.60-545,981.71)	43,041.81 (5,468.27-233,021.32)	0.079
**IL-6**	47.78 (3.71-2,524.54)	116.68 (5.05-2,302.65)	0.335
**IL-17**	25.89 (12-717.60)	44.04 (12-411.25)	0.403
**IFNα2a**	16.4 (5.16-124.31)	24.54 (2.80-42.11)	0.826
**sE-cadherin**	66,766.81 (1,980.38-2,169,304.19)	111,666.7 (2,136.33-1,904,214.72)	0.456
**MMP9**	21,473.07 (124.49-3,560,343.5)	576,234.27 (763.33-2,712,754.25)	0.288

SUPPLEMENTARY FIGURE 1. The levels of cytokines in cervico-vaginal secretions and semen

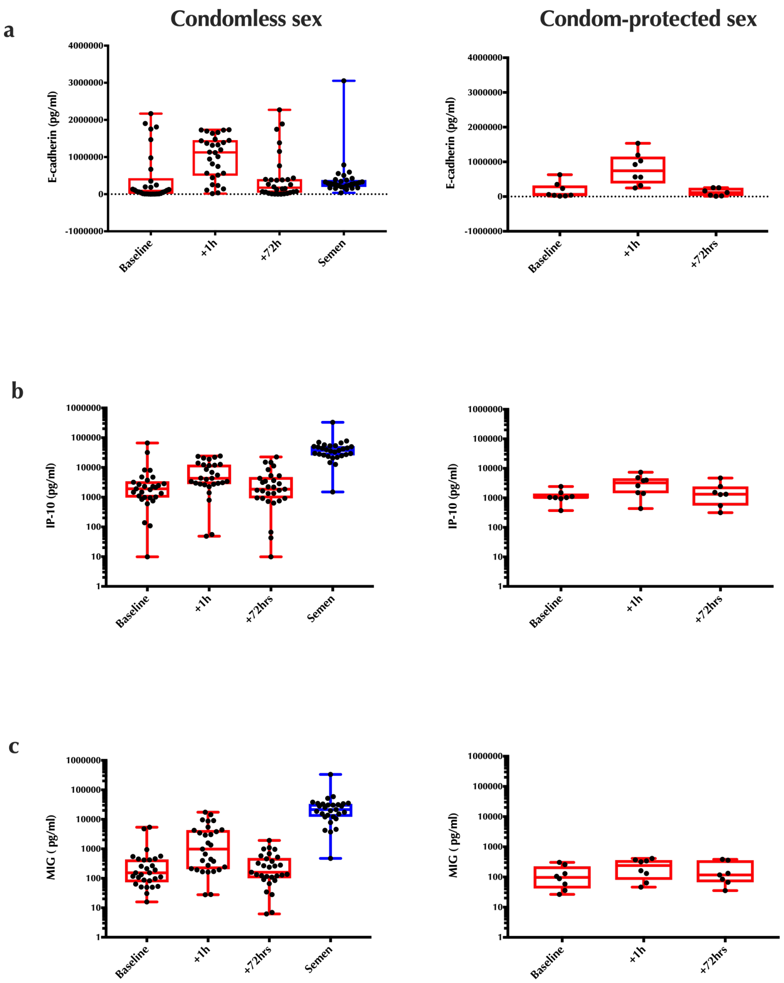

SUPPLEMENTARY FIGURE 1. The levels of cytokines in cervico-vaginal secretions and semen. The level of (a) soluble E-cadherin (pg/ml), (b) IP-10 (pg/ml), (c) MIG (pg/ml), in cervico-vaginal secretion (baseline, 1 hour and 72 hours) after condomless (N=29) and condom-protected sex (N=8), and semen (baseline). The box plots represent the median, with interquartile and minimum to maximum ranges**.** The red box plots represent cervico-vaginal secretions, and the blue box plots represent semen.

SUPPLEMENTARY FIGURE 2. Baseline levels of cytokines in cervico-vaginal secretion of BV+ and BV- women.

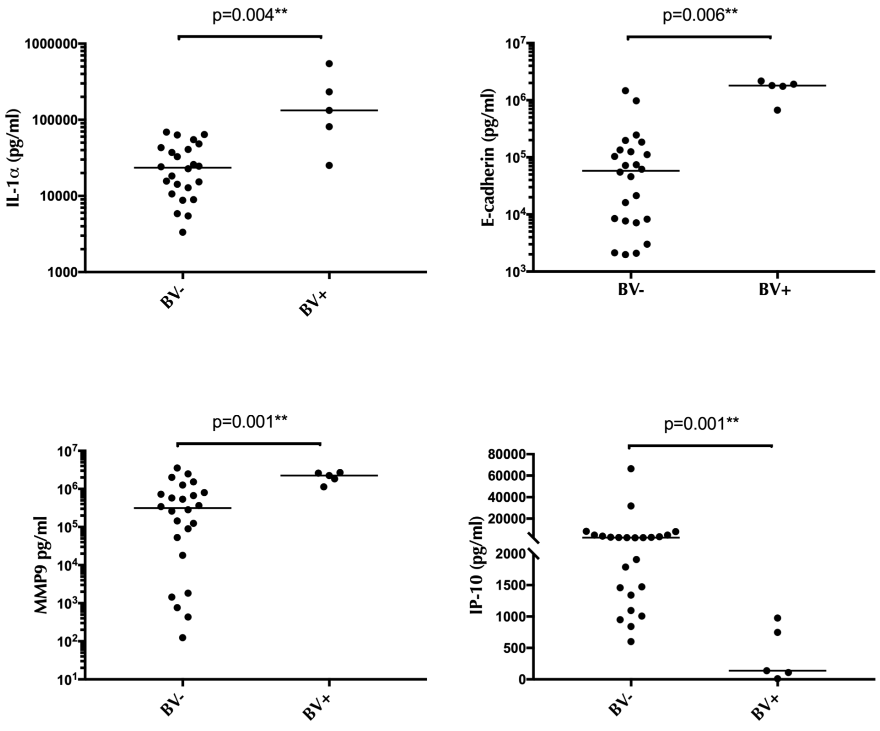

SUPPLEMENTARY FIGURE 2. Baseline levels of cytokines in cervicovaginal secretions of BV+ and BV- women. The level of cervico-vaginal cytokine concentrations is compared between BV+ (N=5) and BV- women (N=24) in condomless group. Statistical comparisons were performed using Mann-Witney U test. BV stands for bacterial vaginosis.

SUPPLEMENTARY FIGURE 3. Gating strategy and representative plots for endocervical activated, CCR5+ and memory CD4+ T cells subsets.

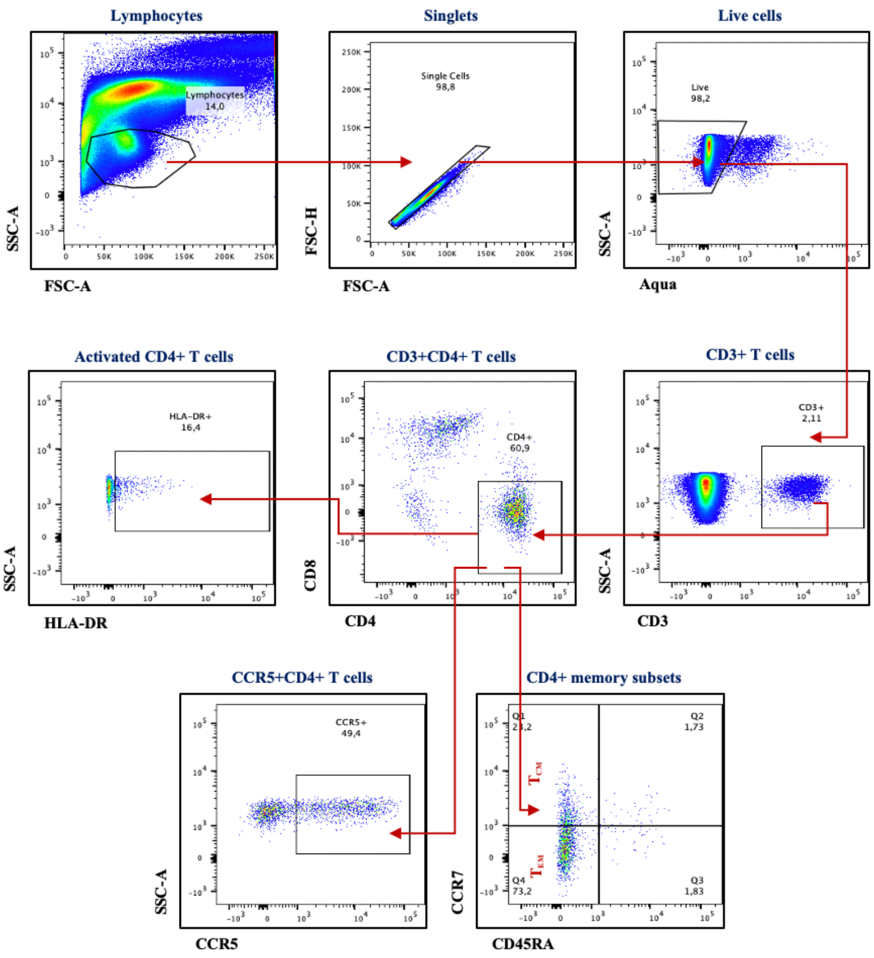

SUPPLEMENTARY FIGURE 3. Gating strategy and representative plots for endocervical activated, CCR5+ and memory CD4+ T cells subsets.

Cells were gated on lymphocytes, singlets, live, CD3+ cells, CD4+ cells, HLA-DR+CD4+ T cells (activated CD4+ T cells), CCR5+CD4+ T cells and CD4+ memory subsets (Central memory: CCR7-CD45RA+; effector memory: CCR7-CD45RA-).

SUPPLEMENTARY FIGURE 4. Gating strategy and representative plots for endocervical Dendritic cells population.

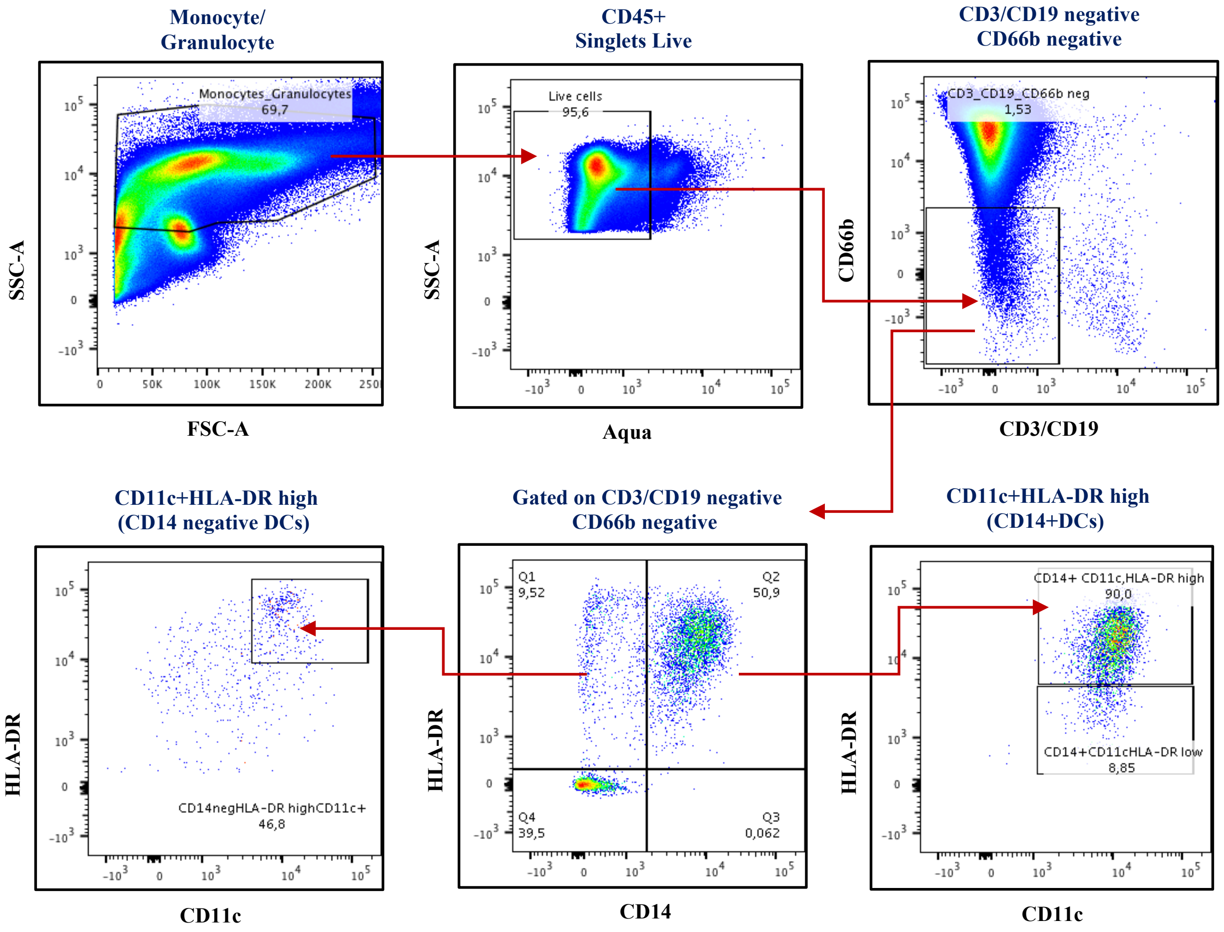

SUPPLEMENTARY FIGURE 4. Gating strategy and representative plots for endocervical Dendritic cells population. Cells were gated on monocytes, CD45+ cells, singlets, CD3/CD19 negative, CD66b negative cells, based on CD14 and HLA-DR expressions two subsets of dendritic cells (DCs): monocyte-derived DCs (CD11c+HLA-Dr highCD14+), CD14- DCs (CD11c+ HLA-DR highCD14-).
