# Supplementary material for: Immune parameters of HIV susceptibility in the female genital tract before and after penile-vaginal sex
Source: Commun Med (Lond). 2022 May 27;2:60. doi: 10.1038/s43856-022-00122-7 (PMC9142516; doi:10.1038/s43856-022-00122-7)
Supplement: Supplementary file 5 — Reporting Summary [file 43856_2022_122_MOESM5_ESM.pdf]

nature research

Corresponding author(s): DBPR COMMSMED-21-0488-T

Last updated by author(s): 22 March 2022

Reporting Summary

Nature Research wishes to improve the reproducibility of the work that we publish. This form provides structure for consistency and transparency in reporting. For further information on Nature Research policies, see our Editorial Policies and the Editorial Policy Checklist.

Statistics

For all statistical analyses, confirm that the following items are present in the figure legend, table legend, main text, or Methods section.

n/a	Confirmed	
☐	☒	The exact sample size (*n*) for each experimental group/condition, given as a discrete number and unit of measurement
☐	☒	A statement on whether measurements were taken from distinct samples or whether the same sample was measured repeatedly
☐	☒	The statistical test(s) used AND whether they are one- or two-sided<br>*Only common tests should be described solely by name; describe more complex techniques in the Methods section.*
☐	☒	A description of all covariates tested
☐	☒	A description of any assumptions or corrections, such as tests of normality and adjustment for multiple comparisons
☐	☒	A full description of the statistical parameters including central tendency (e.g. means) or other basic estimates (e.g. regression coefficient) AND variation (e.g. standard deviation) or associated estimates of uncertainty (e.g. confidence intervals)
☒	☐	For null hypothesis testing, the test statistic (e.g. *F*, *t*, *r*) with confidence intervals, effect sizes, degrees of freedom and *P* value noted<br>*Give P values as exact values whenever suitable.*
☒	☐	For Bayesian analysis, information on the choice of priors and Markov chain Monte Carlo settings
☒	☐	For hierarchical and complex designs, identification of the appropriate level for tests and full reporting of outcomes
☒	☐	Estimates of effect sizes (e.g. Cohen's *d*, Pearson's *r*), indicating how they were calculated

Our web collection on statistics for biologists contains articles on many of the points above.

Software and code

Policy information about availability of computer code

Data collection	N/A
Data analysis	N/A

For manuscripts utilizing custom algorithms or software that are central to the research but not yet described in published literature, software must be made available to editors and reviewers. We strongly encourage code deposition in a community repository (e.g. GitHub). See the Nature Research guidelines for submitting code & software for further information.

Data

Policy information about availability of data

All manuscripts must include a data availability statement. This statement should provide the following information, where applicable:

- Accession codes, unique identifiers, or web links for publicly available datasets
- A list of figures that have associated raw data
- A description of any restrictions on data availability

Data availability. All data generated or analysed in this study will be provided by the corresponding author on request before publication, and will include the link for public access.

Field-specific reporting

Please select the one below that is the best fit for your research. If you are not sure, read the appropriate sections before making your selection.

☒ Life sciences ☐ Behavioural & social sciences ☐ Ecological, evolutionary & environmental sciences

For a reference copy of the document with all sections, see nature.com/documents/nr-reporting-summary-flat.pdf

Life sciences study design

All studies must disclose on these points even when the disclosure is negative.

Sample size	The sample size calculation was based on a small pilot study that assayed inflammatory cytokines in genital secretions . Based on the mean and SD of vaginal IL1 levels, in order to detect a 40% increase after penile vaginal sex with alpha= 0.05 and power= 80% would require a sample size of n=20 couples; in order to permit subanalysis based on male partner circumcision status, we doubled this to n=40 couples.
Data exclusions	Samples with a positive PSA result (indicative of noncompliance with the study protocol) were excluded, as described in the Methods.
Replication	All cytokine measurement were run in duplicate.
Randomization	This was an open label, non-randomized clinical study; the group assignment to condomless sex vs. condom-protected sex was based on participant preferences.
Blinding	Cytokine assays were run by a researcher blinded to the status of participants.

Reporting for specific materials, systems and methods

We require information from authors about some types of materials, experimental systems and methods used in many studies. Here, indicate whether each material, system or method listed is relevant to your study. If you are not sure if a list item applies to your research, read the appropriate section before selecting a response.

Materials & experimental systems

n/a	Involved in the study	
☐	☒	Antibodies
☒	☐	Eukaryotic cell lines
☒	☐	Palaeontology and archaeology
☒	☐	Animals and other organisms
☐	☒	Human research participants
☒	☐	Clinical data
☒	☐	Dual use research of concern

Methods

n/a	Involved in the study	
☒	☐	ChIP-seq
☐	☒	Flow cytometry
☒	☐	MRI-based neuroimaging

Antibodies

Antibodies used

T cell panel (The SECS study)
Antibody-/Fluorochrome /Clone-/Cat Number/Volume per test/Company
CD45-RA /FITC /H100 /304106 /1 µl /BioLegend
CD8 /Percp-Cy5.5/RPA-T8 /45-0088-42 /1 µl /eBioscience
CD103 /B7 APC/ Ber-ACT8 / FIB504 563883 / 551082 /2.5 µl /BD
CD127 /APC-ef780/ ebioRDR5/ 47-1278-42/ 2.5 µl /eBioscience
CD25 /BV421 /M-A251 /562442 /1 µl /BD
LD /Aqua/1 µl
CD4/ BV650 /SK3 /563875 /1 µl /BD
CCR6/ BV711/ 11A9/ 563923 /2.5 µl /BD
CD3/ BV-785/ OKT3 /317330/ 1 µl/ BioLegend
CXCR3/a4-PE/ 1C6 (CXCR3)/9F10/ 557185/555503 BD
CCR5 /PE-CF594 /2D7/ 562456/ 2.5 µl /BD
CCR7/ PE-Cy7/ 3D12/ 557648 /2.5 µl /BD
HLA-DR /BUV-395 /G46-6/ 564040 /2.5 µl /BD
CD69 /BUV-737/ FN50/ 564439 /5 µl /BD

APC panel (The SECS study)
Antibody /Fluorochrome /Clone /Cat Number /Volume per sample/Company
CD14/ FITC /M5E2/ 301804/ 2.5 µl /BioLegend
CD66b /Percp-Cy5.5 /G10F5 /305108/ 2.5µl /eBioscience
BDCA-2/ APC /201A /354206/ 5 µl /BD

CD45/ APC-Fire /HI30/ 304062 /1 µl /eBioscience
CD16 /BV421/ 3G8 /562878/ 1 µl/ BD
LD /Aqua/1 µl
CD83 /BV650 /HB15e /740602/ 2.5 µl /BD
CD11c /BV785 /B-ly6 /740966 /1 µl /BioLegend
CD15 /PE/ HI98 /555402 /5 µl /BD
CD123 /PE-CF594 /7G3 /562391 /5 µl/ BD
CD86 /PE-Cy7 /IT2.2 /305422/ 1 µl /BD
HLA-DR/BUV-395 /G46-6 /2.5 µl /BD
CD3/CD19 /BUV-737 UCHT1/SJ25C1 564307/ 564303 2.5µl BD

Validation

The optimization of Abs concentrations was performed first in blood and then re-validated using endocervical cytobrushes.

Human research participants

Policy information about studies involving human research participants

Population characteristics

The median age of female participants was 22 years (range, 18-33 years) and most (61%) reported contraceptive use other than condoms. Of the male partners, 17 were circumcised and 19 were uncircumcised. The median relationship duration prior to study participation was 18 months (range, 1-96 months). The majority of participants were Asian (41.7%) or white (33.3%).

Recruitment

Couples were recruited into this prospective cohort study through the Women's Health in Women's Hands Community Health Center (WHIWHs) in Toronto. The study protocol was approved by the HIV Research Ethics Board at the University of Toronto. Flyers were posted within the WHIWHs centre and across the University of Toronto St. George campus to recruit couples. Prior to recruitment, the research nurse at WHIWH provided a detailed overview of the study details and requirements to potential participants. At the pre-screening visit, informed consent was obtained from all participants, and they were tested for sexually transmitted infections and pregnancy. Exclusion criteria were infection with HIV1/2, syphilis, Neisseria gonorrhoeae (GC) and/or Chlamydia trachomatis (CT); Ag <16 yrs; pregnancy; any genital ulcers or discharge; irregular bleeding; taking immunosuppressive medications and having taken antibiotics within one month prior to study enrollment

Ethics oversight

The HIV Research Ethics Board at the University of Toronto.

Note that full information on the approval of the study protocol must also be provided in the manuscript.

Flow Cytometry

Plots

Confirm that:

☒ The axis labels state the marker and fluorochrome used (e.g. CD4-FITC).

☒ The axis scales are clearly visible. Include numbers along axes only for bottom left plot of group (a 'group' is an analysis of identical markers).

☒ All plots are contour plots with outliers or pseudocolor plots.

☒ A numerical value for number of cells or percentage (with statistics) is provided.

Methodology

Sample preparation

Peripheral blood mononuclear cells (PBMCs) were isolated by Ficoll-Hypaque density centrifugation at 1500 rpm for 30 min, counted, and washed in R10 medium. One aliquot of one million PBMCs was used for staining of T cell subsets.

Cytobrush processing Protocol:

Cells from two cytobrushes were combined.
1) Vortex the specimen tube (speed on medium) for 10s.
2) Align the two cytobrushes, apply pressure to remove all the mucus.
3) Add 4 ml R10 in such a way as to clean the rim of the tube. Swirl the tube while pouring R10 so all mucus will be removed from the rim of the tube.
4) Vortex the tube for 10s (speed on medium)
5) Pipette up and down and tap gently until all the mucus and cells mix well. Avoid bubbles.
6) Filter endocervical specimen through 100-micron nylon cell strainer (fitted into a 50-ml tube. Add 3 ml R10 to the specimen tube to wash remaining cells from both tube and filter. Collect the entire filtrate in 50 ml tube.
7) Centrifuge at 1700 rpm for 5 min and decant supernatant carefully without disrupting cell pellet.
8) Re-suspend cell pellet by tapping the tube gently. Pipette gently to mix well and avoid bubbles. Divide the cell suspension into 2 equal portions and transfer to FACS tubes for staining

Endocervical cells, PBMCs and whole blood were surface stained with two panels of Abs to characterize various T cell subsets and neutrophils/APC populations. The T cell panel consisted of CD45RA-FITC (BioLegend), CD8- Percp cy5.5 (eBioscience), b7-APC (BD Biosciences), CD127-APCef780 (eBioscience), CD25-BV421(BD Biosciences), CD4-BV650 (BD Biosciences), CCR6-BV711 (BD Biosciences), CD3-BV785 (BD Biosciences), a4-PE (BD Biosciences), CCR5-PE-CF549(BD Biosciences), CCR7-Pe-cy7 (BD Biosciences), HLA-DR-BUV395 (BD Biosciences), CD69-BUV737 (BD Biosciences) and Live/Dead Aqua (Invitrogen). The

neutrophils/APCs panel consisted of CD14-FITC (BioLegend), CD66b-percp-cy5.5 (BioLegend), BDCA-2-APC (BioLegend), CD45-APC-fire (BioLegend), CD16-BV421 (BD Biosciences), CD83-BV650 (BD Biosciences), CD11c-BV785 (BD Biosciences), CD15-Pe (BD Biosciences), CD123- PECF549 (BD Biosciences), CD86-Pe-cy7 (BD Biosciences), HLA-DR- BUV395 (BD Biosciences), CD3/CD19- BUV-737 (BD Biosciences) and Live/ Dead Aqua (Invitrogen).

For each cytobrush sample, all isolated endocervical cells were run through the cytometer, allowing for the endocervical immune cell populations to be quantified as both a proportion (%) of and a total number of cells/cytobrush. In the T cell panel, samples with a CD4+ cell count lower than 10 were excluded from the proportion analysis. In the APC panel, the same exclusion strategy was applied to samples in regard to CD14+ or CD14- cell count.

Instrument

Cells were enumerated using a BD LSR Fortessa X20 flow cytometer (BD Systems).

Software

FlowJo 10.4.1 software (TreeStar, Ashland, OR)

Cell population abundance

No sorting was involved

Gating strategy

All gating strategies for T cell panels were performed based on FMOs controls and for the APC panel based on isotype controls. Representative plots of the gating strategies are presented in the article's supplementary file.

☒ Tick this box to confirm that a figure exemplifying the gating strategy is provided in the Supplementary Information.
